# Supplementary material for: Can an InChI for Nano Address the Need for a Simplified Representation of Complex Nanomaterials across Experimental and Nanoinformatics Studies?
Source: Nanomaterials (Basel). 2020 Dec 11;10(12):2493. doi: 10.3390/nano10122493 (PMC7764592; doi:10.3390/nano10122493)
Supplement: Supplementary file 1 [file nanomaterials-10-02493-s001.pdf]

Table S1. Physicochemical parameters to be determined for different hierarchical tiers with the corresponding characterization techniques [1]. For NM size measurements, readers are referred also to the NanoDefine methods manual [2].

Level/tier	Physicochemical parameter	Suitable characterization techniques
**Chemical substance information (Tier 1)**	Elemental-chemical composition	XRD, XPS, ICP-MS, ICP-OES, SEM-EDX, NMR, MFM, LEIS
	Crystal structure	XRD, EXAFS, HRTEM, electron diffraction, STEM
	Structural defects	HRTEM, EBSD
	Density	DCS, RMM-MEMS, He-pycnometry
	Optical properties	UV-Vis-NIR, PL, EELS-STEM
	Magnetic properties	SQUID, VSM, Mössbauer, MFM, FMR, XMCD, magnetic susceptibility
**Morphology of NM (Tier 2)**	Size (structural properties)	TEM, XRD, DLS, NTA, SAXS, HRTEM, SEM, AFM, EXAFS, FMR, DCS, ICP-MS, UV-Vis, MALDI, NMR, TRPS, EPLS, magnetic susceptibility
	Size distribution	DCS, DLS, SAXS, NTA, ICP-MS, FMR, superparamagnetic relaxometry, DTA, TRPS, SEM
	Shape	TEM, HRTEM, AFM, EPLS, FMR, 3D-tomography
**Surface characteristics (Tiers 3–5)**	Surface composition	XPS, FTIR, NMR, SIMS, FMR, TGA, SANS
	Surface area, specific surface area	BET, liquid NMR
	Chemical state–oxidation state	XAS, EELS, XPS, Mössbauer
	Surface charge	Zeta potential, EPM
	Concentration of surface atoms/ligands/impurities etc.	ICP-MS, UV-Vis, RMM-MEMS, PTA, DCS, TRPS
	Single particle properties	Sp-ICP-MS, MFM, HRTEM, liquid TEM
	Agglomeration state[1]	Zeta potential, DLS, DCS, UV-Vis, SEM, Cryo-TEM, TEM
	Ligand binding/arrangement	XPS, FTIR, NMR, SIMS, FMR, TGA, SANS
	Dispersion of NP in matrices/supports	SEM, AFM, TEM

[1] Although this is an extrinsic property that depends on the surrounding medium, we have kept it in this table of characterization needs as it is an essential parameter for toxicity assessment and is very closely linked with the surface properties of the nanomaterial (NM): "...The surface properties of nanoparticles determine the agglomeration state and the size of the particles under physiological conditions" and "...a comprehensive and accurate characterization of the material under physiological conditions is crucial to correlate the observed biological impact with defined colloidal properties..."[3]

Table S2. Proposal by Gentleman and Chan (2009) for a classification protocol for describing NMs. Reproduced with permission from [4]; Copyright John Wiley and Sons, 2009.

Table 1. Codification protocols for the nanomaterial classification system.

Chemical Class	Size and Shape	Core Chemistry	Ligand Chemistry	Solubility
**XT_1T_2**	**$r(r_e)M_1M_{1b}(m_2)M_3M_4M_5$**	$(Z_1,Z_2,\ldots,Z_n)$	**$[(f_i,f_e)_1;(f_i,f_e)_2;\ldots;(f_i,f_e)_n]$**	**S[log D(pH)]**
**X** 1 if organic/ fullerene (contains no metals)	r = smallest defining dimension in nm<br>r_e = other defining size (if applicable)	**0** if no core	**0** if no ligands	**S**<br>**O** if log D > 1<br>**W** if log D < −1<br>**OW** if −1 < log D < 1
2 if inorganic/ organometallic	**M_1**<br>**B** = ball<br>**H** = polyhedron/faceted<br>**R** = rod/wire<br>**P** = plate/disc/well	list core elements in conventional chemical order; dopants can be included if known	f_i (see Table 2) functional group on inside/ adsorbed to core<br>f_e (see Table 2) outer functional group	indicate log D and pH measurement (if know
**T_1** outermost chemistry<br>**D** = dendrimer<br>**F** = fullerene<br>**L** = liposome<br>**P** = polymer	**M_{1b}** (M_1 value not nec.)<br>**A** = astral (not after B)<br>**I** = irregular			
	m_2 (omit if unknown)<br>B(b), b = # radii:<br>**1** = spheroid; **2** = ellipsoid<br>H(h), h = # faces<br>R(r), r = # barrel faces,<br>**0** = cylinder<br>P(p), p = # sides, **0** = circle<br>A(a), a = # arms	/ indicates inter–core boundary, for example (Cd,Se/Zn,S) is a core/shell	/ indicates multilayer structures, for example $[(f_i,f_e)/(f_i,f_e)]$ is a bilayer for nested structures, only indicate outermost shell	
	**M_3 L** if elongated		bioconjugation $[(f_i,f_e/Bio)]$ or $[(f_i,Bio)]$	
**T_2 N** = nested	**M_4 T** if teethed/ jagged edges		sheet structure, list twice, for example CNT [(Ful,Ful)]	
	**M_5 C** if coiled/helical/ twisted			

Table S3. Overview on safety assessment studies on different morphologies, sizes, and functionalizations of Au NMs (Reproduced with permission from [5,6]; Copyright Royal Society of Chemistry, 2011; Copyright Springer Nature, 2013).

NM Type	Size	Functionalization	Reference
Colloidal Gold (Au nanosphere)	(2 nm)	Quaternary amines	Goodman et al., 2004 [7]
Colloidal Gold (Au nanosphere)	(3 nm)	Tiopronin, TAT-peptides	de la Fuente et al., 2005 [8]
Colloidal Gold (Au nanosphere)	(3.5 nm)	Lysine & poly-L-lysine	Shukla et al., 2005 [9]
Colloidal Gold (Au nanosphere)	(4, 12, 18 nm)	Biotin & CTAB	Conner et al., 2005 [10]
Colloidal Gold (Au nanosphere)	(14 nm)	-	Pernodet et al., 2006 [11]
Colloidal Gold (Au nanosphere)	(15 nm)	Coumarin-PEG-SH	Shenoy et al., 2006 [12]
Colloidal Gold (Au nanosphere)	(20 nm)	BSA & peptides	Tkachenko et al., 2004 [13]
Gold nanorod (GNR Au-Ni)	(200 × 100 nm)	DNA / transferrin	Salem et al., 2003 [14]
Gold nanorod (GNR-65 × 11)	(65 × 11 nm)	CTAB & PEG-SH	Niidome et al., 2006 [15]
Gold nanorod (GNR-65 × 11)	(65 × 11 nm)	CTAB & phosphatidylcholine	Takahashi et al., 2006 [16]
Gold nanoshell			Manju & Sreenivasan, 2010 [17]

An example of the power of identifiers for searching and organising nanosafety data

A simple example of the power of identifiers for NMs are the JRC representative industrial nanomaterial identifiers. A quick overview of these identifiers and ontology mapping is found in a NanoCommons guidance document (https://nanocommons.github.io/specifications/jrc/). Section 3 of this document shows an overview of literature that uses these JRC identifiers in the text, making it easy to find articles about a particular JRC NM. Here, the indexing service is Wikidata [18] allowing us to use Scholia [19,20] to list the information about the JRC NMs (unpublished). For example, on https://scholia.toolforge.org/topic/Q47462008 we can find the literature in which JRCNM01101a is discussed. The guidance also provides the eNanoMapper ontology [21] mappings, allowing to list all TiO_2 JRC nanomaterials. Of course, the list of JRC identifiers is quite limited and not a general solution.

If you want to index identifiers, it must be easy to recognize them. The new JRCNM-based identifiers are therefore better than the older NM-based JRC identifiers. Even then, it can require text mining to extract them. Alternatively, semantic web approaches can be used. The schema.org-based Bioschemas (bioschemas.org) is an ELIXIR-supported open standard that allows providing metadata about the things discussed on webpages [22]. Like schema.org itself, Bioschemas has various topic types it can represent, and *ChemicalSubstance* is most suited for NMs (https://bioschemas.org/profiles/ChemicalSubstance/0.4-RELEASE/, unpublished). The content is commonly added to webpages as a snippet of JSON-LD, and may look like the script shown in Figure S1, which specifies the chemistry of the NM only as ZnO and the size as 152 nm. Thus, it has minimal support for the chemical composition, and a *NInChI* identifier would provide a lot more detail than the above Bioschemas ChemicalSubstance example.

```
{
  "@context" : "https://schema.org",
  "@type" : "ChemicalSubstance" ,
  "name" : "ZnO-152nm" ,
  "identifier" : "JRCNM01101a" ,
  "chemicalComposition" : "ZnO" ,
  "url" : "https://scholia.toolforge.org/topic/Q47462008"
}
```

Figure S1. *ChemicalSubstance* is most suitable Bioschemas format for representing NMs, and adding this text to webpages provides the relevant NM metadata (composition = ZnO; size = 152 nm; Identifier = JRCNM01101a) allowing indexing.

References

1. Mourdikoudis, S.; Pallares, R.M.; Thanh, N.T.K. Characterization techniques for nanoparticles: comparison and complementarity upon studying nanoparticle properties. *Nanoscale* **2018,** *10*, 12871–12934.
2. Friedrich, C.M.; Weigel, S.; Marvin, H.; Rauscher, H.; Wohlleben, W.; Babick, F.; Löschner, K.; Mech, A.; Brüngel, R.; Hodoroaba, V.-D.; Gilliland, D.; Rasmussen, K.; Ghanem, A. The NanoDefine methods manual. Available on line: https://op.europa.eu/en/publication-detail/-/publication/9d60fd79-4244-11ea-9099-01aa75ed71a1/language-en (access on 8 December 2020).
3. Bantz, C.; Koshkina, O.; Lang, T.; Galla, H.J.; Kirkpatrick, C.J.; Stauber, R.H.; Maskos, M. The surface properties of nanoparticles determine the agglomeration state and the size of the particles under physiological conditions. *Beilstein J Nanotechnol.* **2014,** *5*, 1774–1786.
4. Chan, W.C.W.; Gentleman, D.J. A Systematic Nomenclature for Codifying Engineered Nanostructures. Small **2009**, 5, 426–431
5. Khlebtsov, N.; Dykman, L. Biodistribution and toxicity of engineered gold nanoparticles: a review of in vitro and in vivo studies. *Chem. Soc. Rev.* **2011,** *40*, 1647–1671.
6. Kumar, A.; Dhawan, A. Genotoxic and carcinogenic potential of engineered nanoparticles: an update. *Arch. Toxicol.* **2013,** *87*, 1883–1900.
7. Goodman, C.M.; McCusker, C.D.; Yilmaz, T.; Rotello, V.M. Toxicity of Gold Nanoparticles Functionalized with Cationic and Anionic Side Chains. *Bioconjug. Chem.* **2004,** *15*, 897–900.

8. López-Cartes, C.; Rojas, T.C.; Litrán, R.; Martínez-Martínez, D.; de la Fuente, J.M.; Penadés, S.; Fernández, A. Gold Nanoparticles with Different Capping Systems: An Electronic and Structural XAS Analysis. *J. Phys. Chem. B* **2005,** *109*, 8761–8766.
9. Shukla, R.; Bansal, V.; Chaudhary, M.; Basu, A.; Bhonde, R.R.; Sastry, M. Biocompatibility of Gold Nanoparticles and Their Endocytotic Fate Inside the Cellular Compartment: A Microscopic Overview. *Langmuir* **2005,** *21*, 10644–10654.
10. Connor, E.E.; Mwamuka, J.; Gole, A.; Murphy, C.J.; Wyatt, M.D. Gold Nanoparticles Are Taken Up by Human Cells but Do Not Cause Acute Cytotoxicity. *Small* **2005,** *1*, 325–327.
11. Pernodet, N.; Fang, X.; Sun, Y.; Bakhtina, A.; Ramakrishnan, A.; Sokolov, J.; Ulman, A.; Rafailovich, M. Adverse Effects of Citrate/Gold Nanoparticles on Human Dermal Fibroblasts. *Small* **2006,** *2*, 766–773.
12. Shenoy, D.; Fu, W.; Li, J.; Crasto, C.; Jones, G.; DiMarzio, C.; Sridhar, S.; Amiji, M. Surface functionalization of gold nanoparticles using hetero-bifunctional poly(ethylene glycol) spacer for intracellular tracking and delivery. *Int J Nanomedicine* **2006,** *1*, 51–57.
13. Tkachenko, A.G.; Xie, H.; Liu, Y.; Coleman, D.; Ryan, J.; Glomm, W.R.; Shipton, M.K.; Franzen, S.; Feldheim, D.L. Cellular Trajectories of Peptide-Modified Gold Particle Complexes: Comparison of Nuclear Localization Signals and Peptide Transduction Domains. *Bioconjug. Chem.* **2004,** *15*, 482–490.
14. Salem, A.K.; Searson, P.C.; Leong, K.W. Multifunctional nanorods for gene delivery. *Nat Mater.* **2003,** *2*, 668–671.
15. Niidome, T.; Yamagata, M.; Okamoto, Y.; Akiyama, Y.; Takahashi, H.; Kawano, T.; Katayama, Y.; Niidome, Y. PEG-modified gold nanorods with a stealth character for in vivo applications. *J. Control. Release* **2006,** *114*, 343–347.
16. Takahashi, H.; Niidome, Y.; Niidome, T.; Kaneko, K.; Kawasaki, H.; Yamada, S. Modification of Gold Nanorods Using Phosphatidylcholine to Reduce Cytotoxicity. *Langmuir* **2006,** *22*, 2–5.
17. Manju, S.; Sreenivasan, K. 10—Functionalised nanoparticles for targeted drug delivery. In *Biointegration of Medical Implant Materials*, Sharma, C.P., Ed. Woodhead Publishing: Cambridge, UK, 2010; pp. 267–297.
18. Waagmeester, A.S.,G.; Burgstaller-Muehlbacher, S.; Good, B.M., Griffith, M.; Griffith, O.L.; Hanspers, K.; Hermjakob, H.; Hudson, T.S.; Hybiske, K.; Keating, S.M.; et al. Science Forum: Wikidata as a knowledge graph for the life sciences. *eLife Sciences: Computational and Systems Biology* **2020**, doi:10.7554/eLife.52614.
19. Rasberry, L.; Willighagen, E.L.; Nielsen, F.Å.; Mietchen, D. Robustifying Scholia: paving the way for knowledge discovery and research assessment through Wikidata. **2019,** *5*, e35820.
20. Willighagen, E.J., Najko; Nielsen, Finn Årup The EU NanoSafety Cluster as Linked Data visualized with Scholia. In *NSC Newsletter*, figshare, 2018; doi:10.6084/m9.figshare.6727931.v2.
21. Hastings, J.; Jeliazkova, N.; Owen, G.; Tsiliki, G.; Munteanu, C.R.; Steinbeck, C.; Willighagen, E. eNanoMapper: harnessing ontologies to enable data integration for nanomaterial risk assessment. *J. Biomed. Semantics* **2015,** *6*, 10.
22. Michel, F. The Bioschemas Community, Bioschemas & Schema.org: a Lightweight Semantic Layer for Life Sciences Websites. **2018,** *2*, e25836.
